# 

**Published:** 1901-05-11

**Authors:** 


### NOTICE TO CORRESPONDENTS.

All MSS., Letters, Books for Review, and other matters intended for the Editor should be addressed The Editor, "The Hospital," The Hospital Building, 28 & 29 Southampton Street, Strand, W.C.

The Editor cannot undertake to return rejected MS., even when accompanied by stamped directed envelope.

**Notice to Advertisers and Subscribers.**

All Advertisements, Orders for Copies of The Hospital, and Business Communications should be addressed to **THE MANAGER (Not to the Editor),** The Hospital, 28 & 29 Southampton Street, Strand, London, W.C.

The Cost of Subscription, post free, is as follows:—Per week, 2½d.; per quarter, 2s. 8d.; per half-year, 5s. 3d.; per annum, 10s. 6d. Foreign:—Per annum, 15s. 2d., payable, in all cases, in advance.

**NOTICE.—Vol. XXIX. of "The Hospital" (October 1900, to March, 1901), in handsome cloth binding will shortly be on sale at the Office of this Journal, price, 7s. 6d. Cases for binding the Numbers contained in this Volume 1s. 6d. Index to Vol. XXIX., 2½d., post free.**

**The Monthly Part for May is now ready. Price 6d., by post 8d.**

To the Editor of *The Hospital*.

### Scraps and Gleanings.

Under the will of the late Mr. Charles Wheatley, a sum of £500 has passed to the Dewsbury Infirmary.

It is proposed to found a sanatorium for consumption in Ayrshire as a memorial to Queen Victoria.

It has been decided to add a new wing to the Leicester Infirmary, the cost of which will be about £10,000.

The total receipts from the bazaar in aid of the new wing of the Victoria Hospital, Folkestone, amounted to over £900.

At the public meeting held in Bath in support of the Royal United Hospital a sum of £153 was collected or promised.

The estimated cost of the proposed new buildings as planned for the Hip Hospital at Sevenoaks is £10,000, including furnishing and fitting.

The new cottage hospital at Carnarvon which has recently been opened has cost, exclusive of land, £3,750. Of this sum £3,000 had been secured before the opening day.

At the recent quarterly court of Governors of Addenbrooke's Hospital the proposal that electric light should be installed in the hospital at an estimated cost of £400 was agreed to.

The income of the Brotton Cottage Hospital for the past year amounted to £581, as compared with £609 the previous year; and the expenditure was £562 this year as against £545 last year.

The report presented at the annual meeting of the Kent County Ophthalmic Hospital showed that the number of in-patients admitted during the year was 303, the income of the hospital was £1,679, and the expenditure £1,690.

The amount collected during the past year by the Barnsley Hospital Saturday and Sunday organisation amounted to £1,322, the expenses being £41. £1,200 of this amount has been handed to the Beckett Hospital, the balance of £81 being added to next year's account.

During the past year 1,384 in- and 8,608 out-patients were treated at the Bath Royal United Hospital. The ordinary expenditure for the year was £7,000, and extra expenses in connection with drainage works, &c., amounted to £1,369. The total income was £6,198, £812 of which had been received as the result of the collections in workshops and factories, while the church collections realised only £453.

### THE STUDENT OF MEDICINE.

### APPOINTMENTS.

**T. R. Bradshaw,** B.A., M.D.Univ.Dubl., M.R.C.P.Lond., appointed Consulting Physician to the Samaritan Hospital for Women, Liverpool. **Edwin Bramwell,** M.B., appointed a Clinical Assistant (non-resident) in the Edinburgh Royal Infirmary. **Herbert Burland,** L.R.C.P.Lond., M.R.S.C., appointed Certifying Factory Surgeon for the Finedon District of Northants. **L. M. Cairns,** M.B., C.M.Edin., appointed Medical Officer for the Woodhouse District of the Huddersfield Union. **T. P. Cann,** M.B., B.S.Durh., appointed Medical Officer of Health for the Newhaven Urban District. **William Chancellor,** M.B., B.Ch.R.U.I., appointed Certifying Factory Surgeon for the Banbridge District, County Down. **E. F. Clowes,** M.R.C.S., L.R.C.P.Lond., appointed Medical Officer for the Wotton-under-Edge District of the Dursley Union, *vice* B. Simmons, M.R.C.S.Eng., resigned. **H. M. Cory,** M.R.C S., L.R.C.P., appointed Resident Resident Surgical Officer to the Birmingham Children's Hospital. **F. G. Crookshank,** M.D.Lond., appointed Medical Officer of Health for the Barnes Urban District. **E.| Cusse,** M.R.C.S.Eng., appointed Certifying Factory Surgeon for the Broughton District of Hants. **H. H. Davies,** L.R.C.P.I., L.M., appointed Certifying Factory Surgeon for the Cerrig-y-Druidion District of Denbighshire. **W. E. Dawson,** L.R.C.P., L.S.A., &c., appointed House Surgeon to Victoria Hospital, Worksop (The Dukeries). **R. A. Dunn,** M.D.Durh., appointed Medical Officer of Health for the Rural District of Buntingford, Hadham, Hertford, Stansted, and Ware, the Borough of Hertford, and the Urban Districts of Bishop Stortford, Hoddesdon, and Ware. **E. S. B. Eames,** M.R.C.S.Eng., L.R.C.P.Lond., appointed District Medical Officer of the Honiton Union. **D. R. Powell Evans,** L.R.C.P.Lond., M.R.C.S.Eng., L.S.A.Lond., appointed Public Vaccinator for the North Wimbledon District of the Kingston Union. **F. W. Foster,** M.R C.S.Eng., L.R.C.P.Lond., appointed Certifying Factory Surgeon for the Walton-on-Naze District of Essex. **A. Gardiner,** M.B., C.M., appointed Medical Officer for the West District of the Freebridge Lynn Union. **B. Hogan,** L.S.A., appointed Medical Officer for the Bromley District of the Poplar Union. **Thomas D. Lister,** M.D.Lond., M.R.C P., appointed Physician to the City Dispensary. **Andrew Mair,** M.B., Ch.B.Glas., appointed Assistant to the Professor of Pathology, Dundee University College, St. Andrews University. **J. H. Maund,** M.R.C S., L.R.C.P.Lond., appointed Medical Officer of Health for the Newmarket Urban District. **T. Perrin,** M.R.C.S., L.R.C.P., appointed Assistant Medical Officer to the Lambeth Infirmary. **R. W. C. Pierce,** B.Sc., M.B.Lond., D.P.H.Camb., appointed Medical Officer of Health to the Guildford Rural and Woking Urban District Councils, and Medical Officer to the Guildford, Godalming, and Woking Joint Hospital Board. **R. G. Pollock,** M.R.C.S., L.R.C.P.Lond., appointed Certifying Factory Surgeon for the Warlingham District of Surrey. **M. H. Raper,** M.D.Lond., M.R.C.S.Eng., appointed Certifying Factory Surgeon for the Great Wakering District of Essex. **Edward Davies Rees,** M.R.C.S.Eng., L.R.C.P.Lond., L.S.A., appointed Medical Officer of the Newtown and Llanidloes Union Workhouse at Caersws, Montgomery. **J. Sproull,** M.B.Glasg., appointed Medical Officer of Health for the Luddenden Foot Urban District. **Walter H. Swaffield,** L.R.C.P. and S.Edin., appointed a Clinical Assistant (non-resident) in the Edinburgh Royal Infirmary.

THE MENOPAUSE AND ITS DISORDERS.

With Chapters on Menstruation. By A. D. LEITH NAPIER, M.D., M.R.C.P., late Editor of the *British Gynæcological Journal*. Royal 8vo. cloth gilt, illustrated by a series of Original Photo-micrographs and many Illustrations in the Text, 7s. 6d. net.

"Of great practical use to the general practitioner as well as to those more specially engaged in gynæcological work."—*The Hospital.*

THE SCIENTIFIC PRESS, LTD.,
28 and 29 Southampton Street, Strand, London, W.C.

